# A comparison of FLAG-Ida and daunorubicin combined with clofarabine in high-risk acute myeloid leukaemia: data from the UK NCRI AML17 Trial

**DOI:** 10.1038/s41375-018-0148-3

**Published:** 2018-06-06

**Authors:** A K Burnett, R K Hills, O J Nielsen, S Freeman, A Ali, P Cahalin, A Hunter, I F Thomas, N H Russell

**Affiliations:** 1 Formerly Cardiff University School of Medicine, Cardiff, UK; 2 Centre for Trials Research, Cardiff University, Cardiff, UK; 3 Department of Haematology, Rigshospitalet, Copenhagen, Denmark; 4 Institute of Immunology and Immunotherapy, University of Birmingham, Birmingham, UK; 5 Department of Haematology, Castle Hill Hospital, Hull, UK; 6 Department of Haematology, Blackpool Victoria Hospital, Blackpool, UK; 7 Department of Haematology, Leicester Royal Infirmary, Leicester, UK; 8 Department of Haematology, Nottingham University Hospitals NHS Trust, Nottingham, UK

**Keywords:** Acute myeloid leukaemia, Chemotherapy

Gradual but definite progress has been made in the treatment of younger adults with acute myeloid leukaemia (AML) [1–3], except those 25–30% with high-risk disease. For them the best approach is a myeloablative allogeneic transplant; however, even the transplant option provides only a 30% chance of cure, and has been limited to younger patients who have a donor. The point at which a patient is recognised to be at high risk is usually after the first course of induction treatment has been given when the most useful prognostic data is to hand.

These patients represent an important unmet therapeutic need for which there is no specific standard of care. The challenge is three-fold. First, for those who will go forward to transplant improving the pre-transplant chemotherapy could reduce the post-transplant relapse rate. Second, better treatment could deliver more patients to transplant who otherwise might relapse before reaching transplant. Third, better treatment is needed for patients for whom a transplant is not available.

For relapsed patients the FLAG-Ida (fludarabine/cytosine arabinoside (ara-C)/granulocyte-colony stimulating factor (G-CSF) and idarubicin) is widely used, although it has not been subjected to a randomised assessment. In our Medical Research Council (MRC) AML15 Trial, FLAG-Ida given for the first two treatment courses had a significantly superior anti-leukaemia effect [4]. It therefore appeared logical to continue with FLAG-Ida as consolidation for patients who were identified as high risk. We chose as the comparative treatment to replace ara-C, in a daunorubicin/ara-C combination, with an alternative nucleoside, clofarabine, which has demonstrated activity in adverse risk patients [5–9]. Following a successful feasibility study combining daunorubicin with clofarabine, we decided to prospectively compare this combination with FLAG-Ida in high-risk patients.

The AML17 protocol (ISRCTN55675535) was designed to include untreated de novo or secondary AML and high-risk myelodysplastic syndrome (defined as >10% marrow blasts at diagnosis). The overall treatment plan has been described elsewhere [10] and was conducted in accordance with the Declaration of Helsinki, sponsored by the Cardiff University, and approved by the Wales Research Ethics Committee 3. The age range was between 16 and 61 years, although older patients were permitted if deemed suitable for the potential randomisations. Three hundred and eleven patients were randomised after having received the first induction course, which could have been ADE (Ara-C/daunorubicin/etoposide) alone (*N* = 39) or with gemtuzumab ozogamicin (GO) 3 mg/m^2^ (*n* = 29) or 6 mg/m^2^ (*n* = 30) or daunorubicin/Ara-C (DA) with GO 3 mg/m^2^ (*N* = 35) or 6 mg/m^2^ (*N* = 22) with the daunorubicin dose being 60 mg/m^2^ (*n* = 78) or 90 mg/m^2^ (*n* = 78). GO was given on day 1. The patients were designated as high risk by our validated weighted score (based on cytogenetics, gender, white count, secondary disease, older age, and failure to achieve at least a reduction in marrow blasts to <15%, or to 50% of blasts at diagnosis) [11, 12]. Consenting patients were randomised (2:1) to receive up to three courses of DClo (daunorubicin 50 mg/m^2^ on days 1, 3 and 5 and clofarabine 20 mg/m^2^ days 1–5) or FLAG-Ida (fludarabine 30 mg/m^2^ days 2–6, ara-C 2 g/m^2^ days 2–6, G-CSF 263 µg days 1–7, idarubicin 8 mg/m^2^ days 4–6) with the intention of going to allogeneic transplant if feasible. The end points of interest were the number of patients delivered to transplant and overall survival (OS) whether transplanted or not.

Toxicity (haematologic recovery times and non-haematologic toxicity) were scored using National Cancer Institute Common Toxicity Criteria, version 3 and resource use was collected. All end points were defined according to the revised International Working Group Criteria [13]

With 315 patients, the trial had 80% power to detect an improvement in survival from 30 to 45%, and an improvement in the number of patients receiving a transplant from 40 to 57%.

All analyses are by intention to treat. Categorical end points (e.g. complete remission (CR) rates) were compared using Mantel-Haenszel tests, giving Peto odds ratios (ORs) and confidence intervals. Continuous/scale variables were analysed by non-parametric (Wilcoxon's rank-sum) tests. Time-to-event outcomes were analysed using the log-rank test, with Kaplan–Meier survival curves. ORs/hazard ratios (OR/HRs) <1 indicate benefit for DClo. Survival percentages are at 5 years except for patients censored at transplant which is at 4 years. Median follow-up is 47.4 months (range 3.1–74.7 months). In addition to overall analyses, exploratory analyses were performed stratified by the randomisation stratification parameters and other important variables, with suitable tests for interaction. Because of the well-known dangers of subgroup analysis, these were interpreted cautiously.

Between September 2009 and October 2012, of 1583 patients in the AML17 Trial, 468 (30%) became eligible for the high-risk intervention, of whom 311 patients (66%) entered the randomisation in whom the OS was 31% at 5 years, which was not significantly different from the 157 eligible, but un-randomised, patients (25%; *p* = 0.18). The characteristics and responses are shown in Table 1. The proportion of *FLT3-*mutated patients is low because such patients entered the lestaurtinib randomisation in the trial [10]. Eighty-nine patients (29%) were >60 years. Minimal/measurable residual disease (MRD) information, as previously defined [14], was available in 88 patients. There was a significant trend towards increasing high risk with age. One hundred and sixty-nine patients (54%) were already in CR or complete remission with incomplete count recovery (CRi) (DClo 54%; FLAG-Ida 55%) and a further 52 had confirmed CR/CRi after first course (representing 32% of DClo patients not known to be in remission at randomisation, and 47% of FLAG-Ida patients). Seventeen percent of DClo and 14% of FLAG-Ida had resistant disease. In the DClo arm, the number of courses given was—0: 1%; 1: 38%; 2: 47%; 3: 14%, and in the FLAG-Ida arm was—1: 56%; 2: 38%; 3: 6%.

**Table 1:** Patient characteristics and outcomes

	**DClo**	**FLAG-Ida**	**OR/HR, 95% CI**	***p*** **Value**
Number randomised	207	104		
Age group (years)				
15–29 (16%)	6 (3%)	7 (7%)		
30–39 (20%)	17 (8%)	8 (8%)		
40–49 (19%)	29 (14%)	14 (13%)		
50–59 (29%)	95 (46%)	46 (44%)		
60+ (37%)	60 (29%)	29 (28%)		
Gender				
Female	66 (32%)	35 (34%)		
Male	141 (68%)	69 (66%)		
Type of disease				
De novo	147 (71%)	74 (71%)		
Secondary	38 (18%)	20 (19%)		
High-risk MDS	22 (11%)	10 (10%)		
Performance status				
0	149 (72%)	75 (72%)		
1	50 (24%)	24 (23%)		
2	5 (2%)	2 (2%)		
3	3 (1%)	3 (3%)		
4	0	0		
Induction treatment				
ADE (NR)	11 (5%)	6 (6%)		
ADE	14 (7%)	8 (8%)		
ADE + GO3	20 (10%)	9 (9%)		
ADE + GO6	20 (10%)	10 (10%)		
DA + GO3	23 (11%)	12 (12%)		
DA + GO6	15 (7%)	7 (7%)		
DA60	52 (25%)	26 (25%)		
DA90	52 (25%)	26 (25%)		
Cytogenetics				
Favourable	0	0		
Intermediate	102 (49%)	49 (47%)		
Adverse	105 (51%)	55 (53%)		
NK	0	0		
FLT3-ITD				
WT	195 (94%)	96 (92%)		
Mutant	4 (2%)	1 (1%)		
Not known	8 (4%)	7 (7%)		
NPM1				
WT	173 (84%)	87 (84%)		
Mutant	25 (12%)	9 (9%)		
Not known	9 (4%)	8 (8%)		
MRD status post C1				
−ve	21 (10%)	8 (8%)		
+ve	43 (21%)	16 (15%)		
Not in CR	65 (31%)	31 (30%)		
Not known	78 (38%)	49 (47%)		
MRD status post C2				
CR/CRi, MRD −ve	20 (11%)	12 (13%)		MRD −ve vs. MRD +ve vs. no CR, *p* = 0.08
CR/CRi, MRD +ve	29 (15%)	18 (20%)		
CR/CRi, MRD unk	93 (49%)	49 (53%)		
Not in CR	47 (25%)	13 (14%)		
Not known	18	12		
ORR (CR + Cri)	83%	86%	1.24 (0.66–2.34)	0.5
CR	68%	72%	1.23 (0.74–2.05)	0.4
CRi	15%	13%		
30-day mortality	2%	4%	0.61 (0.15–2.45)	0.5
60-day mortality	9%	10%	0.95 (0.44–2.06)	0.9
5-year OS	26%	44%	1.40 (1.05–1.86)	0.02
4-year OS censored at SCT	15%	28%	1.27 (0.87–1.85)	0.2
5-year CIR	51%	39%	1.38 (0.95–2.01)	0.09
5-year CIDCR	24%	17%	1.45 (0.83–2.51)	0.19
5-year RFS	25%	44%	1.40 (1.03–1.91)	0.03

Number within parentheses indicates the percentage of non-APL patients in each age group who entered the high-risk randomisation expressed as a proportion of all patients entering the AML17 Trial

*CR* complete remission, *APL* acute promyelocytic leukaemia, *AML* acute myeloid leukaemia, *FLT3* fms-like tyrosine kinase 3, *ITD* internal tandem duplication, *MRD* minimal/measurable residual disease, *CRi* complete remission with incomplete count recovery, *NPM1* nucleophosmin member 1, *NK* natural killer, *WT* wild type, *OR* odds ratio, *CI* confidence interval, *OS* overall survival, *ORR* overall response ratio, *SCT* stem cell transplant, *CIR* cumulative incidence of relapse, *MDS* myelodysplastic syndrome, *CIDCR* cumulative incidence of death in remission, *RFS* relapse-free survival

Overall 175 (56%) received a transplant and was not different between the arms (DClo 58%: FLAG-Ida 53%) (OR 0.83 (0.52–1.34), *p* = 0.4). For DClo 74 received a matched myeloablative transplant (20 siblings, 54 MUDs) and FLAG-Ida 33 (15 siblings, 18 MUDs). Reduced intensity allografts were given to 37 (14 siblings, 23 MUDs) in the DClo arm, and 12 (3 siblings, 9 MUDs) in the FLAG-Ida arm. There were an additional 31 transplants reported of other or unknown type (DClo 21, FLAG-Ida 10), giving an overall transplant rate of 120/207 (58%) vs. 55/104 (53%) (OR 0.81 (0.51–1.31), *p* = 0.4).

The cumulative incidence of relapse (CIR), relapse-free survival (RFS) and OS at 5 years for DClo vs. FLAG-Ida were 51 vs. 39% (HR 1.38 (0.95–2.01), *p* = 0.09); 25 vs. 44% (HR 1.40 (1.03–1.91), *p* = 0.03); and 26 vs. 44% (HR 1.40 (1.05–1.86), *p* = 0.02) (Table 1 and Fig. 1a–c). There were no significant interactions between treatment effect and age (*p* = 0.5) or cytogenetics (*p* = 0.3). When outcomes are censored at the time of transplant, the effect estimate for survival was similar (4-year survival 15 vs. 28%; HR 1.27 (0.87–1.85), *p* = 0.2) (Fig. 1d). However, RFS post transplant in first remission was worse in patients receiving transplant following DClo compared with following FLAG-Ida (39 vs. 69%; HR 1.96 (1.19–3.21), *p* = 0.008).

**Fig. 1:**
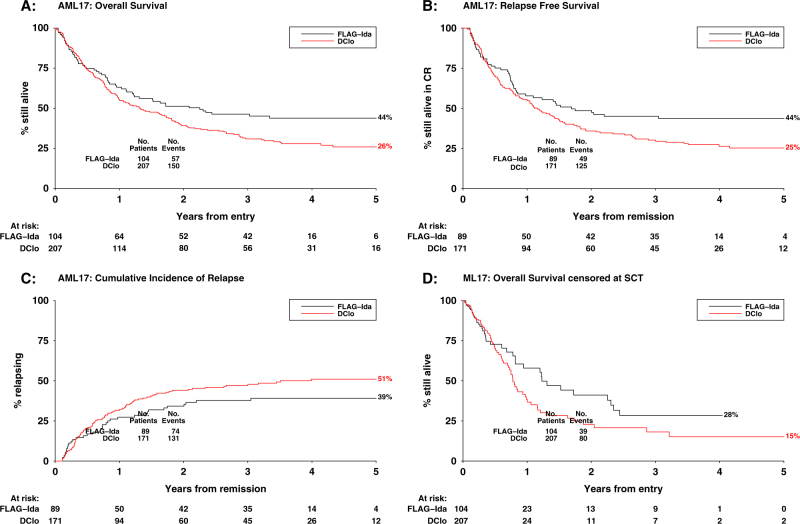
Outcome data. **a** Overall survival, **b** relapse-free survival, **c** cumulative incidence of relapse and **d** survival censored at stem cell transplantation

FLAG-Ida was significantly more myelosuppressive than DClo. Neutrophil recovery to 1.0 × 10^9^/l was longer (40 vs. 32 days) (*p* = 0.005) as was platelets recovery to 100 × 10^9^/l (61 vs. 45 days) (*p* = 0.16), resulting in a greater requirement for red cells (10.7 vs. 6.9) and platelets (12.9 vs. 7.1), days on antibiotics (19.3 vs. 10.6) and days in hospital (median 31 vs. 23) (*p* < 0.0001 for each). The non-haematological toxicities observed were similar after course 2 of treatment.

Minimal (measurable) residual disease was undertaken by flow cytometry in a parallel lab study. The sensitivity was 1 × 10^4^ and the results were not conveyed to investigators. There was no difference between the arms at randomisation post course 1 of chemotherapy in the proportion entering with or without MRD information/being MRD +ve or MRD −ve. However, after the first course of randomised treatment, significantly fewer DClo patients were in confirmed morphological remission (75 vs. 86% of those with data, *p* = 0.04), and there was evidence of worse MRD response overall (*p* = 0.08), although the proportions of the 79 patients in CR with MRD information who were MRD −ve was similar between arms (41 vs. 40%, *p* = 0.9).

In our previous experience, about 40% of high-risk patients got to transplant, which resulted in an RFS of 39%. Although FLAG-Ida is widely used in relapsed disease, it has not been assessed by randomised comparison. Here 84% of patients achieved CR or CRi as the best response with no differences between the arms. Although FLAG-Ida did not deliver more patients to allograft (43 vs. 48% with DClo), OS was significantly better than with DClo, partly because of the better RFS after transplant (69 vs. 39%). In patients who were not already in remission on entering the randomisation FLAG-Ida did not deliver more patients to transplant. Although the observations were limited, the response by MRD status was not different between the arms.

We conclude that the FLAG-Ida was superior in high-risk patients, despite not delivering more patients to transplant. However, the resultant prolonged cytopenia can cause unpredictable logistic difficulties in scheduling the transplant, so checking the marrow status 10–14 days after the chemotherapy and proceeding to transplant irrespective of count recovery may be optimal. Survival also was significantly better when transplant was not undertaken (44 vs. 28%), although there are likely to be major selection biases in play in making such a comparison.

## Electronic supplementary material

Supplementary material
