# Supplementary material for: A comparison of FLAG-Ida and daunorubicin combined with clofarabine in high-risk acute myeloid leukaemia: data from the UK NCRI AML17 Trial
Source: Leukemia. 2018 Jun 6;32(12):2693–7. doi: 10.1038/s41375-018-0148-3 (PMC6286330; doi:10.1038/s41375-018-0148-3)

Supplementary Material: The Risk Score[15]

Using Cox regression on the MRC AML10 and AML12 trials, for non-APL adults under the age of 50, a forward selection model was derived for overall survival from remission, with the following candidate variables:

- Age
- WBC
- Performance status
- Sex
- de Novo/Secondary
- Cytogenetics (Using Grimwade classification favourable/intermediate/adverse)
- Platelets
- BM blasts
- Response after course 1 (CR/PR/NR)
- Height
- Weight

The level of significance to enter the model was set at p=0.05.

In order of entry to the model, the variables which make up the index are:

Variable	Estimate	χ^2	p-value
Cytogenetics	0.65082	102.7	<0.0001
Age	0.01325	29.16	<0.0001
Status post C1	0.19529	18.50	<0.0001
WBC	0.00169	11.92	0.0006
Male sex	0.16994	8.01	0.005
Secondary	0.22131	4.03	0.04

The index is therefore:

0.01325*age (in years) + **0.16994***sex (1=male, 0=female) + **0.22131***diagnosis (1=de novo, 2 secondary) + **0.65082***cytogenetics (1=favourable, 2=intermediate, 3 adverse) + **0.19529***status post C1 (1=CR, 2=PR, 3=NR) + **0.00169*** WBC (x10^9/l)

and the distribution of patients in AML10,12 by index is:

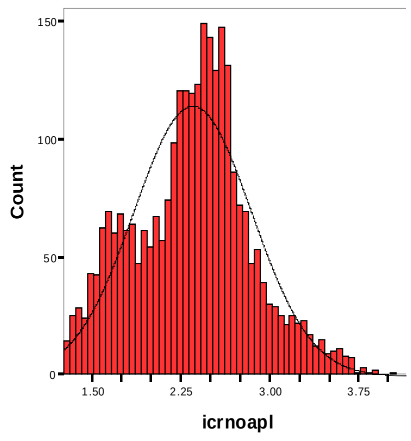

Taking into account the apparent bimodality of the curve, patients with an index of 2 or below were deemed good risk, and the data were arbitrarily divided at the 75th centile between standard and poor risk as this would capture nearly all patients previously deemed high risk using the MRC index used in AML12. Survival from CR in AML10,12 according to the risk groups was as follows:

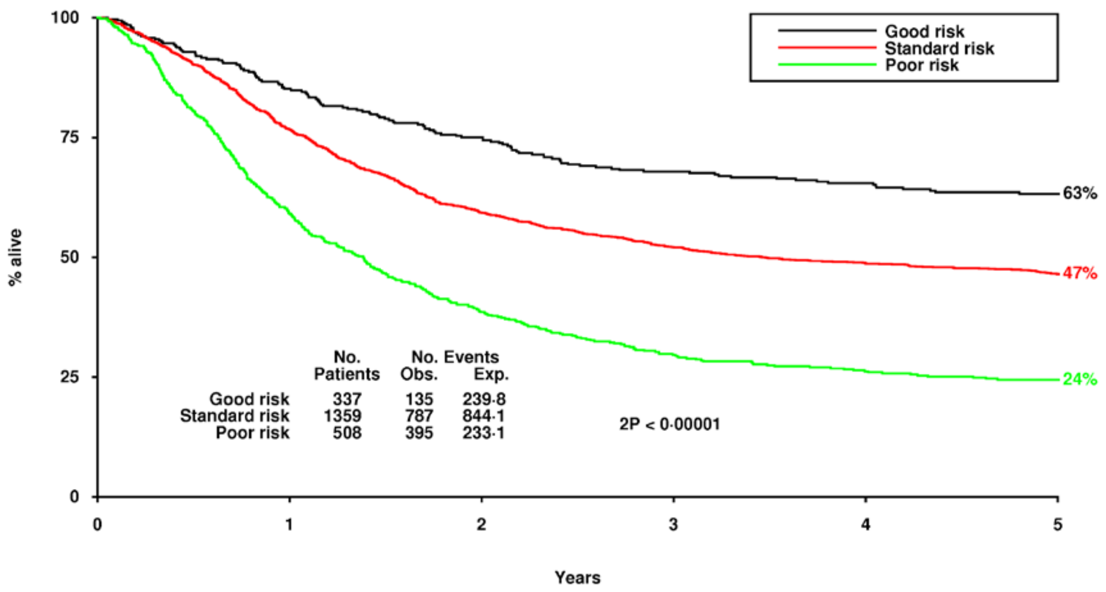

The index was validated on data from MRC AML15 Trial:

MRC AML 15 (no APL): Survival from CR by post CR risk group

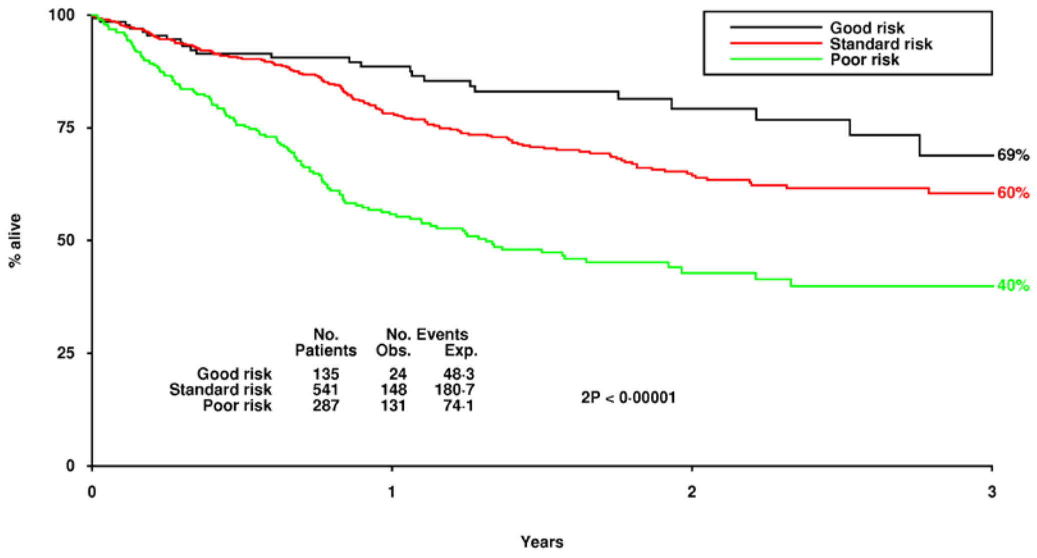

Supplement: Supplementary file 1 — Supplementary material [file 41375_2018_148_MOESM1_ESM.pdf]
